# An annual land cover dataset for the Baltic Sea Region with crop types and peat bogs at 30 m from 2000 to 2022

**DOI:** 10.1038/s41597-024-04062-w

**Published:** 2024-11-18

**Authors:** Vu-Dong Pham, Farina de Waard, Fabian Thiel, Bernd Bobertz, Christina Hellmann, Duc-Viet Nguyen, Felix Beer, M. Arasumani, Marcel Schwieder, Jörg Hartleib, David Frantz, Sebastian van der Linden

**Affiliations:** 1 Institute of Geography and Geology, University of Greifswald, Partner in the Greifswald Mire Centre, Friedrich-Ludwig-Jahn-Str. 16, 17489 Greifswald, Germany; 2 Interdisciplinary Centre for Baltic Sea Region Research (IFZO), University of Greifswald, 17489 Greifswald, Germany; 3 Thünen Institute of Farm Economics, Bundesallee 63, 38116 Braunschweig, Germany; 4 Geography Department, Humboldt-Universität zu Berlin, Unter den Linden 6, 10099 Berlin, Germany; 5 Geoinformatics – Spatial Data Science, Trier University, Behringstraße 21, Trier, 54296 Germany

**Keywords:** Environmental impact, Ecosystem ecology

## Abstract

We present detailed annual land cover maps for the Baltic Sea region, spanning more than two decades (2000–2022). The maps provide information on eighteen land cover (LC) classes, including eight general LC types, eight major crop types and grassland, and two peat bog-related classes. Our maps represent the first homogenized annual dataset for the region and address gaps in current land use and land cover products, such as a lack of detail on crop sequences and peat bog exploitation. To create the maps, we used annual multi-temporal remote sensing data combined with a data encoding structure and deep learning classification. We obtained the training data from publicly available open datasets. The maps were validated using independent field survey data from the Land Use/Cover Area Frame Survey (LUCAS) and expert annotations from high-resolution imagery. The quantitative and qualitative results of the maps provide a reliable data source for monitoring agricultural transformations, peat bog exploitation, and restoration activities in the Baltic Sea region and its surrounding countries.

## Background & Summary

Land use/land cover (LULC) products are valuable for assessing the status of remaining natural habitats and determining the degree of human pressure on natural ecosystems. Over the past few decades, the availability of openly and globally accessible remote sensing data has fuelled various studies to map LULC over extended areas and time periods^1^. In Europe, the CORINE Land Cover (CLC^2^) is a well-established and comprehensive LULC product that provides thematic LULC maps of roughly 44 land cover (LC) classes across multiple years. The CLC product has set up a standard for subsequent studies in Europe that followed a similar LC classification scheme to produce finer spatial resolution and denser time-series LULC maps^3–8^.

However, one of the drawbacks of CLC and its derivatives is the lack of detailed information on croplands. For example, the CLC classification scheme contains 44 LC classes, but for agricultural land, which accounts for more than 42% of LC in Europe^9^, it does not further differentiate. At the same time, detailed information on agricultural land use enables monitoring of the spatial distribution of crop types, the analysis of crop sequences, and the assessment of the composition of the agricultural landscape as a whole, which is crucial in the context of biodiversity^10^. Crop sequence information is also proving useful given the rapid changes that agricultural practices in Europe have undergone in adapting to climate change over the last few decades^11^. Although there have been attempts to map crop types at both national and continental scales^12–14^, they are often only available for single or short periods. LULC products with crop type information of large regions and over decadal periods are still generally scarce.

Located in the center of Europe, the Baltic Sea region (BSR) has witnessed a similar pace of change in agriculture as the rest of the continent^15^. In addition to that, the BSR has also experienced the degradation of natural peat bogs and other mires through various activities such as peat harvesting, or draining peatlands to be used as cropland, grassland, or forestry. Since the beginning of 2000, peat extraction in bogs has been estimated at up to 1.2 million t/year in Estonia, and roughly 0.5–0.7 million t/year in Latvia and Lithuania^16^. The degradation of peatlands due to mining activities results in adverse environmental impacts across the BSR, including losses in carbon sequestration^17^, potential and biodiversity^18^ and water pollution^19^. In addition, degraded peatlands are important carbon sources, with peat decomposition under aerobic conditions causing large amounts of greenhouse gas (GHG) emissions^20,21^. Many factors contribute to the rates of GHG emissions, e.g., management on grassland and organic soils^22^, land use changes^23^ and agricultural practice^24^, and foremost the water tables depth of drained peatlands^20^. Hence, annual monitoring of peat bog exploitation in the BSR is essential to inform policy-making and facilitate the conservation of its associated ecosystems, particularly in response to the challenges posed by the climate crisis. This could be achieved by interpreting LULC maps with information of exploited and unexploited peat bogs, as well as the land use on drained peatland. So far, there are only a few LULC datasets in Europe that contain information on natural peat bogs and their exploitation. For example, datasets such as Natura 2000^25^ or Coastal Zone^26^ provide LC maps with this information, but only within the limited boundaries of natural reserves and coastal areas. In addition, these maps only exist for specific years, while spatially continuous annual products, which would allow to identify rewetting activities following peat harvesting, for example, are still lacking. Such maps would be helpful for monitoring the success of restoration and efficacy of restoration strategies^27^.

To bridge this gap, we here present the Baltic Sea Region Land Cover *Plus* (BSRLC+), the first set of annual land cover maps at 30 m resolution of the BSR over two decades (2000–2022), containing detailed information on crop types and peat bog extractions.

## Methods

### Study area

**Fig. 1:**
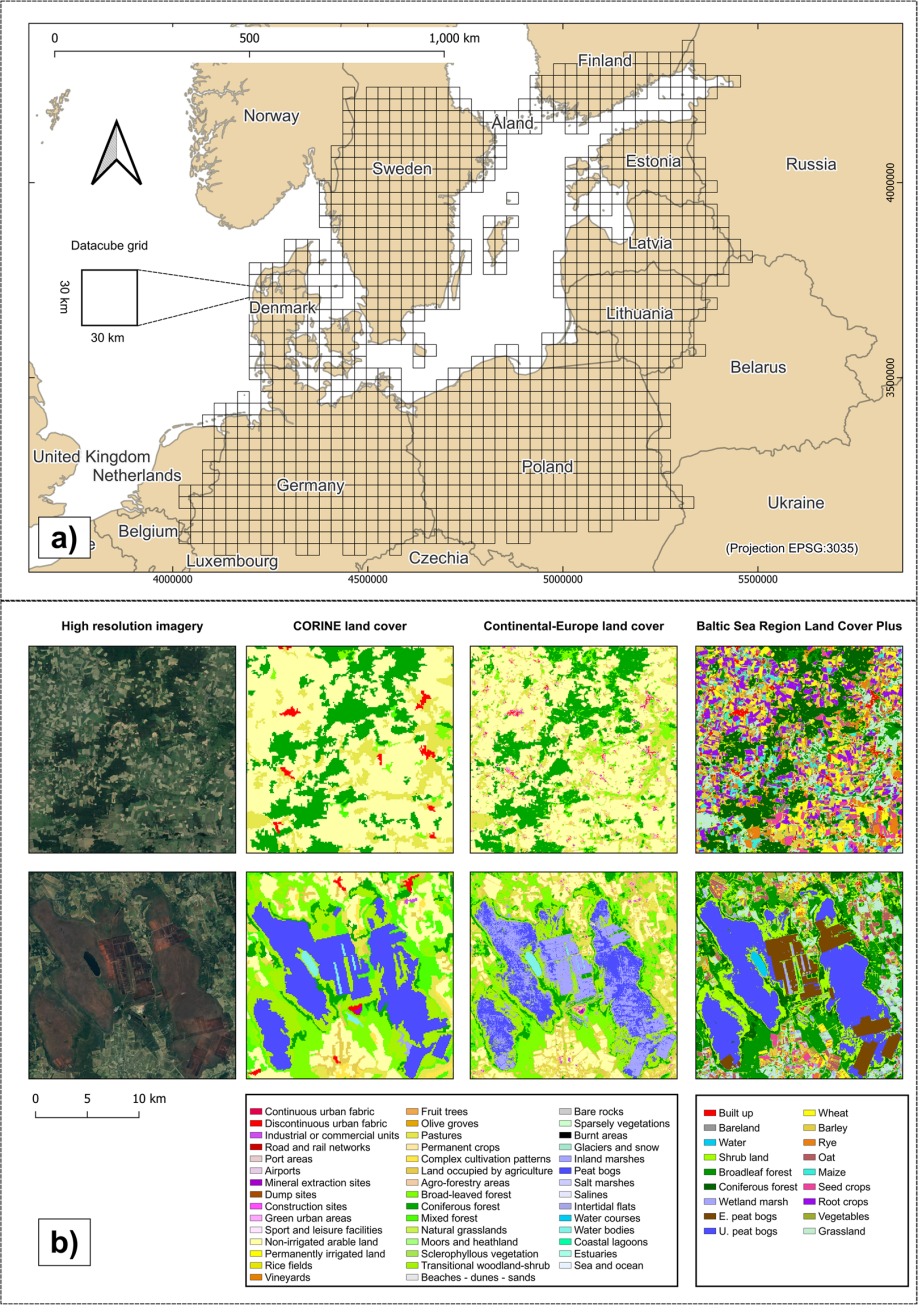
(**a**) The Baltic Sea region; (**b**) Thematic details of CORINE Land Cover^2^ and Continental Europe Land Cover^6^ (44 classes) compared to Baltic Sea Region Land Cover *Plus* (18 classes) maps for 2018; upper example: an area in Germany (center coordinate 52.89 N, 10.85E) dominated by agricultural land, which is oversimplified by existing LC products, whereas our maps reveal the diverse land use in agriculture; bottom example: our map distinguishes unexploited and exploited peat bogs in Estonia (center coordinate 58.54 N, 24.37E). High resolution images are taken from Google Earth.

We mapped the BSR, here defined as land masses bordering the Baltic Sea without the Gulf of Bothnia. The area covers a total area of 1,143,000 km^2^ and spans over 9 countries (Fig. 1). It fully covers Denmark, Estonia, Latvia, and Lithuania, northern parts of Germany and Poland and southern Sweden and Finland. From Russia the Kaliningrad exclave as well as the coastline between Finland and Estonia are covered. All islands within the geographic extent are also included, as well as coastal waters within the respective image tiles of the covered land areas.

### Overall workflow

**Fig. 2:**
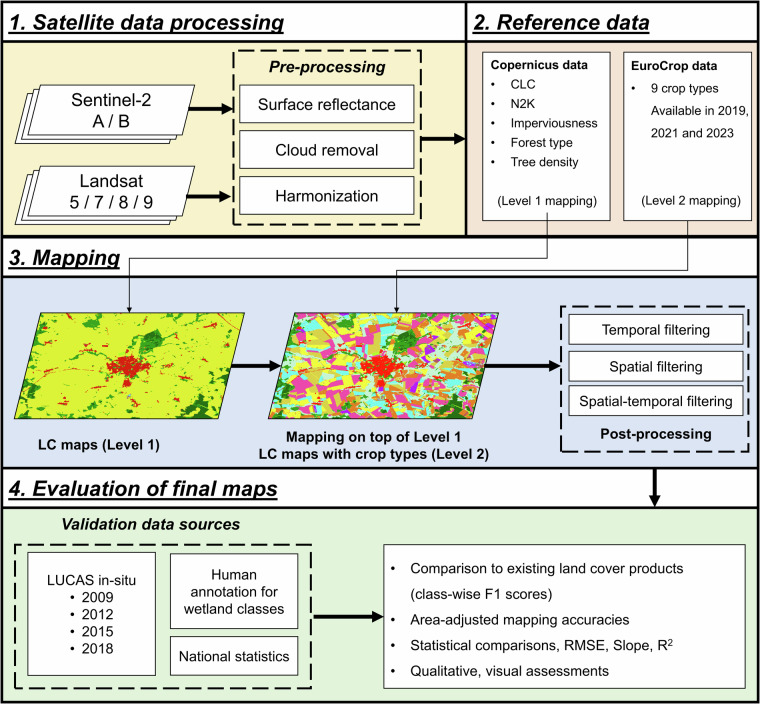
Overall workflow.

To create the maps, we structured a workflow (Fig. 2) that incorporates multiple processes:

- Satellite data processing: We downloaded and processed all available Landsat and Sentinel-2 imageries over more than two decades (from 2000 to 2022). This includes estimating surface reflectance, cloud removal and data harmonization.
- Reference data: We collected various existing, open LC datasets and applied different sampling strategies to sample reference LC data to train the machine learning model.
- Mapping: We used deep learning classification and performed hierarchical mapping to predict first level maps (Level 1) containing eight general LC types, followed by detailed prediction of LC maps with crop types and wetland types (Level 2) on top of the Level 1 results. The final maps contain eighteen LC types. For each level, we applied different temporal and spatial filtering methods to remove noise.
- Evaluation of final maps: We assessed map accuracies using independent *in-situ* reference LC data and national statistics, using various quantitative metrics, as well as qualitative assessments by comparing with very high-resolution imageries.

### Land cover classes

We mapped annual LC using a hierarchical approach, with the low-level map (Level 1) containing eight general LC types: Built-up, Bareland, Water, Shrubland, Coniferous forest, Broadleaf forest, Wetland, Cropland and Grassland. The final high-level map (Level 2) provides more details by separating the Wetland class into Wetland marsh, Exploited peat bog, and Unexploited peat bog as well as separating Cropland and Grassland into: Wheat, Barley, Rye, Oat, Maize, Seed crops, Root crops, Dry pulses and vegetable, Grassland. In Level 2 the Grassland class comprises all areas of the open landscape that are not used as arable land. This includes meadows and pastures as well as (semi-) natural grasslands. In summary, we mapped a total of eighteen LC classes, the nomenclature for each class is shown in Table 1.

**Table 1:** Land cover hierarchy and nomenclature.

Land cover Level 1	Land cover Level 2	Nomenclature
Built-up		Land that is covered by building structures and transport networks. Excluding mining areas
Bareland		Open spaces with little or no vegetation throughout the entire mapping period. Beaches, sand dunes, bare rocks, cliffs, including bare surfaces such as open mines.
Water		Permanent open water courses, lakes, reservoirs, sea and ocean
Shrubland		Heathland, moorland, and areas in transitional woodland-shrubland.
Coniferous forest		Woody vegetation, principally trees, where coniferous species predominate.
Broadleaf forest		Woody vegetation, principally trees, where broadleaf species predominate.
Wetland	Wetland marsh	Inland freshwater marshes and inland salt marshes.
	Exploited peat bog	Open exploited peat-producing wetlands that are not greatly affected by lakes, sea water or water from water courses.
	Unexploited peat bog	Open unexploited peat-producing wetlands that are not greatly affected by lakes, sea water or water from water courses.
Crop land and grassland.	Wheat, Barley, Rye; Oat, Maize, Seed crops, Root crops, Dry pulses and vegetable, Grassland	Arable land, permanent crops, heterogeneous agricultural areas. The Grassland class contains meadows, pastures, natural and semi-natural grassland.

### Reference land cover data

To support supervised machine learning classification over large space and time, diverse and extensive training data are essential. For non-crop-related LC classes, we used existing LC maps, datasets, and remote sensing spectral indices, combined with rule-based filtering for semi-automated data collection. The datasets, mostly including those from the Copernicus Land Monitoring Service (CLMS, https://land.copernicus.eu/), provided detailed quantitative and categorical maps useful for sampling reference LC types (see Table 2).

**Table 2:** Reference LC data for training classification.

Data name	Description	Data source
Imperviousness layers	Pan-European level in the spatial resolution of 10 m and 100 m the sealing density in the range from 0% to 100% for the 2018 reference year, 20 m and 100 m for the 2006, 2009, 2012, and 2015 reference years.	https://land.copernicus.eu/en/products/high-resolution-layer-imperviousness
Tree cover density layers	Pan-European level in the spatial resolution of 10 m and 100 m the level of tree cover density in a range from 0% to100% for the 2018 reference year, 20 m and 100 m for the 2012 and 2015 reference years.	https://land.copernicus.eu/en/products/high-resolution-layer-tree-cover-density
Forest type layers	Pan-European level in the spatial resolution of 10 m and 100 m a forest classification for three thematic classes (all non-forest areas/broadleaved forest/coniferous forest) with the agricultural/urban trees removed for the 2018 reference year, 20 m and 100 m for the 2012 and 2015 reference years.	https://land.copernicus.eu/en/products/high-resolution-layer-forest-type
CORINE land cover (CLC)	Pan-European CORINE Land Cover inventory for 44 thematic classes for the 2006, 2012, 2018 reference years. The dataset has a Minimum Mapping Unit (MMU) of 25 hectares (ha) for areal phenomena and a Minimum Mapping Width (MMW) of 100 m for linear phenomena and is available as vector and as 100 m raster data.	https://land.copernicus.eu/en/products/corine-land-cover
Natura 2000 (N2K)	Detailed land cover and land use information for 55 thematic classes in selected Natura2000 sites for the 2006, 2012, 2018 reference years. The dataset has a Minimum Mapping Unit (MMU) of 0.5 ha and a Minimum Mapping Width (MMW) of 10 m and is available as vector data.	https://land.copernicus.eu/en/products/n2k
Spectral temporal metrics (STM)	Spectral temporal metrics (STM, e.g., minimum, maximum, median, percentiles, etc.) of Normalized Difference Vegetation Index (NDVI^28^) and Normalized Difference Water Index (NDWI^29^)	See in Remote sensing data section

All datasets that we used (Table 2) were rasterized or resampled to a 30 m resolution (using cubic interpolation for continuous data and nearest neighbour interpolation for categorical data) to be comparable to our satellite remote sensing data (see Remote sensing data section). From the selected dataset, for each interested LC class, we applied multiple rule-based methods to acquire large amounts of reference LC points with high confidence. All sampled points were considered consistent (invariant in LC type) during the period from 2006 to 2018, in details:

- Built-up: Imperviousness dataset (2006, 2009, 2012, 2015, 2018) were used. A 5 × 5 (pixels) focal filter runs across the dataset in all five years. The center pixels were selected as Built-up if they satisfy all the following criteria: (1) All surrounding pixels (24 pixels) have imperviousness values > 20% in all years; (2) The center pixel has more than 50% of imperviousness in all five years.
- Bareland: Spectral temporal metrics (STM) of Normalized Difference Vegetation Index (NDVI^28^) and Normalized Difference Water Index (NDWI^29^), and Imperviousness dataset (2006, 2009, 2012, 2015, 2018) were used. Based on our analysis on spectral profiles, Bareland pixels were selected if they satisfy all of the following criteria: (1) 90^th^ percentile NDVI value is lower than 0.3 throughout 2006–2018 (to filter out pixels with vegetation signal); (2) 90^th^ percentile NDWI value is lower than 0 throughout 2006–2018 (to filter out pixels dominated by water); (3) All imperviousness values = 0 in 5 years (2006, 2009, 2012, 2015, 2018) (to filter out pixels dominated, or close to built-up areas).
- Water: Spectral temporal metrics (STM) of NDWI and NDVI, and Imperviousness dataset (2006, 2009, 2012, 2015, 2018) were used. Based on our analysis on spectral profiles, Water pixels were selected only if they satisfy all the following criteria: (1) 10^th^ percentile NDWI value is greater than 0.3 throughout 2006–2018 (to ensure the pixels are dominated by permanent water); (2) 90^th^ percentile NDVI value is lower than 0.3 throughout 2006–2018 (to filter out pixels with strong vegetation signal); (3) All imperviousness values = 0 in 5 years (2006, 2009, 2012, 2015, 2018).
- Shrubland: CLC dataset (2006, 2012, 2018), N2K dataset (2006, 2012, 2018), Tree Density dataset (2012, 2015, 2018) and Imperviousness (2006, 2009, 2012, 2015, 2018) were used. Shrubland pixels were selected if they satisfy all the following criteria: (1) Both CLC and N2K contains one of these classes: Moors and heathland, Sclerophyllous vegetation and Transitional woodland-shrub in all three years 2006, 2012 2018; (2) Tree density values in the pixels < 30% in three years 2012, 2015, 2018; (3) All imperviousness values = 0 in 5 years 2006, 2009, 2012, 2015, 2018.
- Coniferous forest: CLC dataset (2006, 2012, 2018), forest type dataset (2006, 2012, 2018), and tree density dataset (2012, 2015, 2018) were used. A 5 × 5 focal filter runs across all datasets. The center pixels were selected as Coniferous forest if they satisfy all the following criteria: (1) All pixels (25 pixels) are classified as coniferous forest in all three years 2006, 2012, 2018 in the CLC dataset; (2) All pixels (25 pixels) are classified as coniferous forest in all three years 2012, 2015, 2018 in the forest type dataset; (3) All pixels (25 pixels) have tree density values > 75% in all three years 2012, 2015, 2018.
- Broadleaf forest: CLC dataset (2006, 2012, 2018), forest type dataset (2006, 2012, 2018), and tree density dataset (2012, 2015, 2018) were used. A 5 × 5 focal filter runs across all datasets. The center pixels were selected as Broadleaf forest if they satisfy all the following criteria: (1) All pixels (25 pixels) are classified as broadleaf forest in all three years 2006, 2012, 2018 in the CLC dataset; (2) All pixels (25 pixels) are classified as broadleaf forest in all three years 2006, 2012, 2018 in the forest type dataset; (3) All pixels (25 pixels) have tree density values > 75% in all three years 2012, 2015, 2018.
- Wetland marsh: CLC dataset (2006, 2012, 2018) and N2K dataset (2006, 2012, 2018) were used. A 5 × 5 focal filter runs across all datasets. The center pixels were selected as Wetland marsh if they satisfy all the following criteria: (1) All pixels (25 pixels) are classified as inland marsh or salt marsh in all three years 2006, 2012, 2018 in the CLC dataset; (2) All pixels (25 pixels) are classified as inland marsh or salt marsh in all three years 2006, 2012, 2018 in the N2K dataset.
- Exploited peat bog: CLC dataset (2006, 2012, 2018) and N2K dataset (2006, 2012, 2018) were used. A 5 × 5 focal filter runs across all datasets. The center pixels were selected as Exploited peat bog if they satisfy all of the following criteria: (1) All pixels (25 pixels) are classified as peat bog in all three years (2006, 2012, 2018) from in the CLC dataset; (2) All pixels (25 pixels) are classified as exploited peat bog in all three years (2006, 2012, 2018) in the N2K dataset.
- Unexploited peat bog: CLC dataset (2006, 2012, 2018) and N2K dataset (2006, 2012, 2018) were used. A 5 × 5 focal filter runs across all datasets. The center pixels were selected as Unexploited peat bog if they satisfy all of the following criteria: (1) All pixels (25 pixels) are classified as peat bog in all three years (2006, 2012, 2018) from in the CLC dataset; (2) All pixels (25 pixels) are classified as unexploited peat bog in all three years (2006, 2012, 2018) in the N2K dataset.
- Crop land and grassland: CLC dataset (2006, 2012, 2018), N2K dataset (2006, 2012, 2018), Tree density (2012, 2015, 2018), and Imperviousness (2006, 2009, 2012, 2015, 2018) were used. A 5 × 5 focal filter runs across all datasets. The center pixels were selected as Cropland and grassland if they satisfy all the following criteria: (1) Both CLC and N2K contains one of these classes in all three years (2006, 2012, 2018): Irrigated and non-irrigated arable land, Managed grassland (Pasture), Natural grassland; (2) All pixels (25 pixels) have Tree density values = 0% in all three years 2012, 2015, 2018; (3) All pixels (25 pixels) have Imperviousness values = 0% in all five years (2006, 2009, 2012, 2015, 2018).

We sampled up to 10,000 training pixels per class, and each is considered invariant in LC type during 2006–2018. This way, for each sample, we can derive multi-annual spectral profiles from remote sensing data, which enhance the temporal transferability of supervised classifications (see details in Classification section).

For crop type reference data, we used the EuroCrop dataset^30^, which includes harmonized crop polygons from sixteen European countries. We used all available datasets that overlapped with the BSR. From the reference data statistics, we defined nine major crop types in the area: Wheat; Barley; Rye; Oat; Maize; Seed crops; Root crops; Dry pulses and vegetables; and Grassland (see Table 3 for nomenclature).

**Table 3:** Crop types reference from EuroCrop dataset^30^.

LC class	Nomenclature	Training data (all following data sources are provided by EuroCrop GitHub repository: https://github.com/maja601/EuroCrops; latest access 09.09.2024)
Wheat	Winter/Spring soft wheat; Spring/Winter durum hard wheat; Buckwheat.	• 2019: – Denmark: https://landbrugsgeodata.fvm.dk/ • 2021: – Sweden: https://djur.jordbruksverket.se/swedishboardofagriculture.4.6621c2fb1231eb917e680002462.html – Estonia: https://inspire-geoportal.ec.europa.eu/overview.html?view=thematicEuOverview&theme=none – Lithuania: https://www.geoportal.lt/geoportal/nacionaline-mokejimo-agentura-prie-zemes-ukio-ministerijos#savedSearchId=%7B772172A4-6719-48BD-8DDC-5DEEFB27DE74%7D&collapsed=true – Latvia: https://www.lad.gov.lv/lv/atbalsta-veidi/platibu-maksajumi/lauku-registrs-un-karte/lauku-registra-dati/ – Germany (Lower Saxony): https://sla.niedersachsen.de/landentwicklung/LEA/ • 2023: – Germany (Brandenburg): https://geobroker.geobasis-bb.de/gbss.php?MODE=GetProductInformation&PRODUCTID=996f8fd1-c662-4975-b680-3b611fcb5d1f
Barley	Winter/Spring barley.	
Rye	Winter/Spring rye	
Oat	Winter/Spring oat.	
Maize	Grain maize and green silo maize	
Seed crops	Winter/Spring/Summer rapeseed; Flax seed; Oil seed; Sunflower.	
Root crops	Potatoes; Sweet Potatoes; Sugar beet.	
Dry pulses, vegetable	Legumes; Beans; Chickpeas; Lentils; Sweet lupin; Peas; Fresh vegetables; Strawberry.	
Grassland	Pasture, meadow, grassland.	

We rasterized all crop reference data to a 30 m resolution that aligned with our remote sensing data (see Remote sensing data section). Next, in each year where crop data was available (2019, 2021 and 2023), we randomly sampled up to 50,000 training pixels per class. As a result, a total of around 2 million crop reference pixels were used for training.

### Remote sensing data

**Fig. 3:**
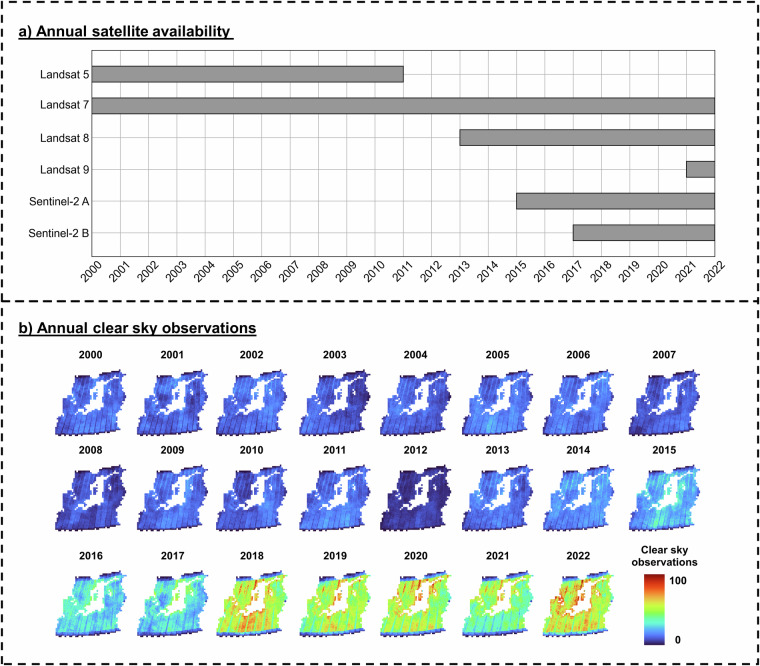
(**a**) Annual satellite availability in the study area. (**b**) Annual clear sky observations (CSO) from 2000 to 2022.

We downloaded all available remote sensing satellite scenes covering the BSR from 2000 to 2022 (with cloud cover less than 75% per scene) of Landsat 5 TM (LS5); Landsat 7 ETM+ (LS7); Landsat 8 OLI (LS8); Landsat 9 OLI+ (LS9) provided by United States Geological Survey (USGS, https://earthexplorer.usgs.gov/), and Sentinel-2A (S2A) and Sentinel-2B (S2B) provided by the Copernicus Open Access Hub (https://scihub.copernicus.eu/maintenance.html). Annual satellite availability is shown in Fig. 3a. In the study area, we limited the map coverage to land area only and intentionally excluded all tiles fully and permanently covered with water. This greatly reduced the physical space for remote sensing data storage as well as compute processing units.

All satellite data were harmonized and processed to Level-2 surface reflectance using the Framework for Operational Radiometric Correction for Environmental monitoring (FORCE^31^). Six reflectance bands were used: Red, Green, Blue, Near-Infrared, Shortwave-Infrared 1 and Shortwave-Infrared 2. We also included three additional indices: Normalized Difference Vegetation Index (NDVI^28^), Normalized Difference Water Index (NDWI^29^) and Soil-Adjusted Vegetation Index (SAVI^32^). All bands were processed at 30 m resolution, whereas higher resolution bands (from Sentinel-2 data) were resampled to the target resolution with FORCE using an approximated point spread function. The raster data were reprojected to ETRS89-extended/LAEA Europe (EPSG:3035) and divided into a regular 30 × 30 km grid (see Fig. 1). We derived annual clear sky observations (CSO) to provide an overview of data density per year (Fig. 3b).

### Classification

**Fig. 4:**
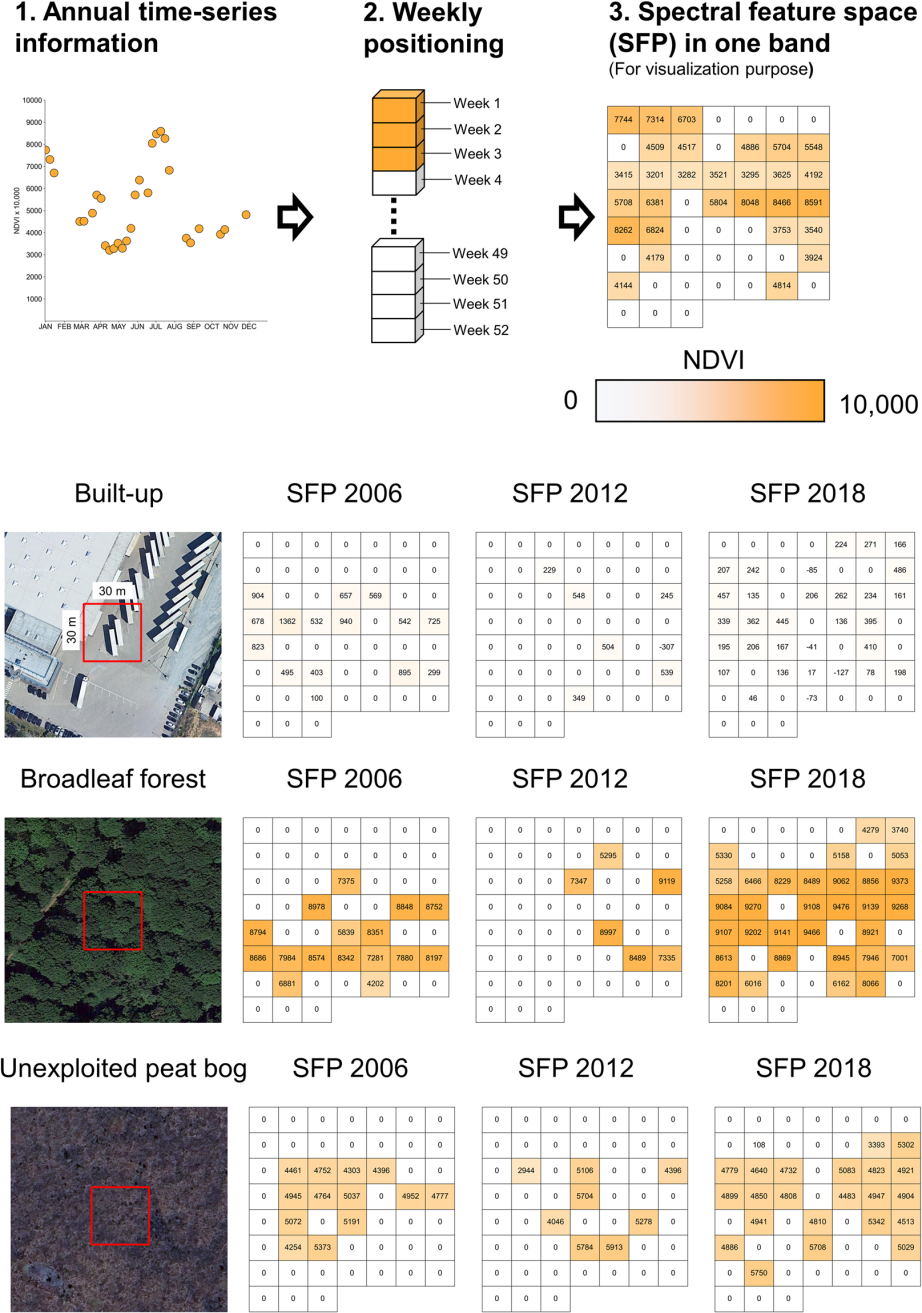
(Top): Temporal encoding^33^ process converts annual time-series information to 52 feature spaces in one band (NDVI is used as an example, scaling by 10,000). (Bottom): Examples of spectral NDVI feature spaces for three LC types: built-up, broadleaf forest and unexploited peat bog in three different years (2006, 2012, 2018). Red boxes in the high resolution image chips represent a 30 m × 30 m pixel over a corresponding land cover type.
- The input data (52 time-steps x 9 bands) is then used to train the 1-Dimensional Convolutional Neural Network (1D-CNN) classifier. Here, the 1D convolution layers are applied to the temporal dimension of the input (Fig. 5), followed by max pooling layers and fully connected layers for estimating land cover type probabilities. Details of the network architecture is provided in Supplementary File 1.

The availability of remote sensing data varied greatly over years (Fig. 3). Therefore, when using temporal information as input data, it is often required to use aggregation methods to create equidistant feature spaces to support machine learning models^1^. However, Pham *et al*.^33^ demonstrated that most aggregation methods often transfer poorly when facing irregular temporal data, especially when mapping crop types. The authors proposed a generalized method for capturing annual time-series information called Temporal Encoding. This method involves filling a 365-feature data structure with clear observations, placing each observation in a position corresponding to its acquisition date. For days without clear observations, a blank value (0) is assigned. This way, the encoded input data is neither compressed nor extrapolated while remaining the consistent input feature length. The method has been shown to be highly robust even when the temporal data density varies between training and mapping data. In this study, we adapted the methods from Pham *et al*.^33^ with some slight alterations:

- We used weekly encoding: In each satellite band, we created an array with 52 time-steps, representing 52 weeks of the year. For each time-step, all clear observations of every week (7 days) are averaged and positioned to their corresponding week. Weeks that do not have any clear observations are assigned with values of 0. Using weekly encoding allows the input features 7-times lighter compared to daily encoding (365 time-steps) used in Pham *et al*.^33^, while still preserving the detailed LC phenology information (Fig. 4). In this study, we used 9 bands (6 spectral bands and 3 indices), making a total of 486 input features (52 time-steps x 9 bands) for the classification model. Visualizations of the spectral features space of different land cover types for different time periods are shown in Fig. 4

**Fig. 5:**
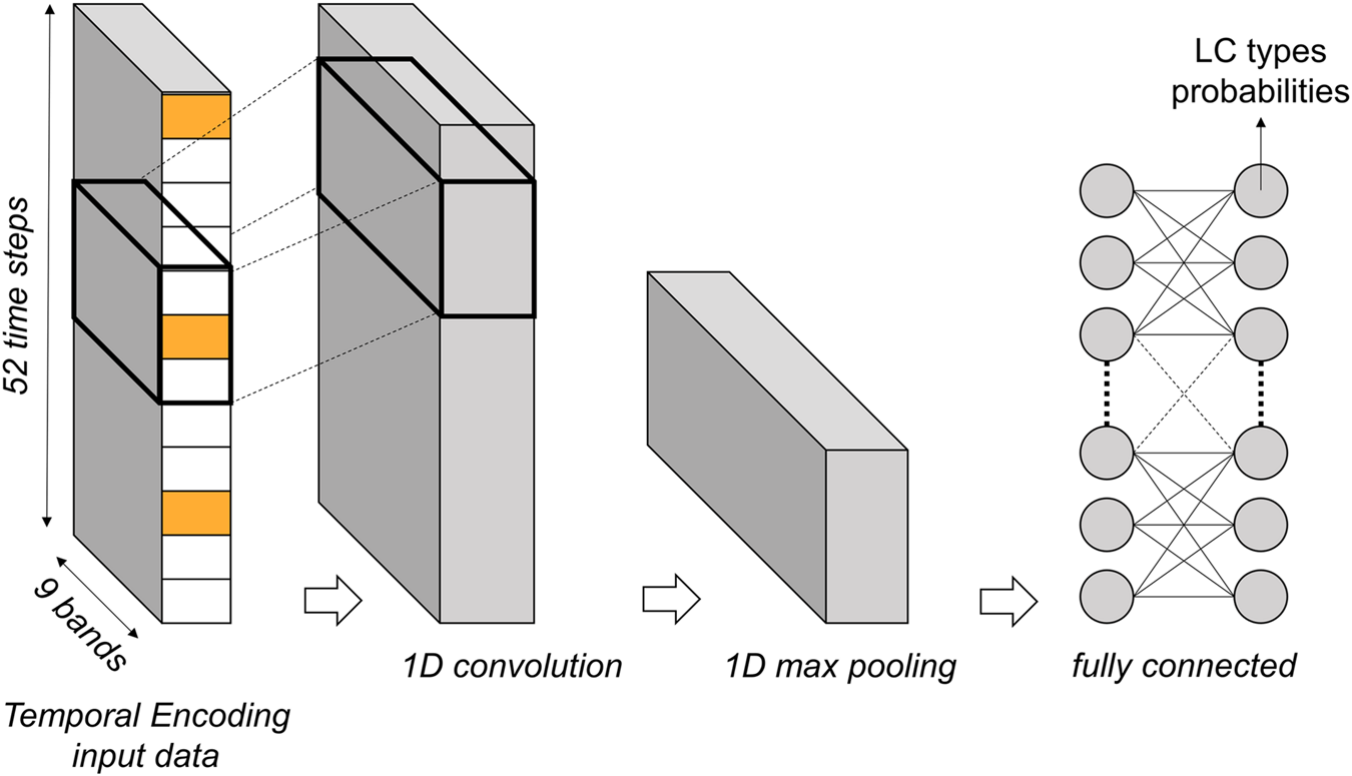
Simplified 1-Dimensional Convolutional Neural Network (1D-CNN) architecture for land cover classification with temporal encoding input. Details of the network architecture is provided in Supplement File 1.
- During the training process, we applied two data augmentation methods Random Observation Selection and Random Day Shifting proposed in Pham *et al*.^33^. These methods are used to simulate the temporal data sparsity and phenological shifts. Incorporating Temporal Encoding and data augmentations have been shown to greatly improve the transferability of the deep learning model, allowing to transfer the model trained with data from recent years to past years^33^.

To improve the mapping of agricultural and wetland areas, we used a hierarchical classification scheme (Fig. 2). In the first step (Level 1) the following general land cover types are differentiated: Built-up, Bareland, Water, Shrubland, Coniferous forest, Broadleaf forest, Wetland, Cropland and Grassland. Subsequently, the high-level more detailed classification (Level 2) was performed on top of level 1 maps. Here the Wetland class is further distinguished into: Wetland marsh, Exploited peat bog and Unexploited peat bog; and the classes Cropland and Grassland into: Wheat, Barley, Rye, Oat, Maize, Seed crops, Root crops, Dry pulses and vegetable, and Grassland.

### Post processing

**Fig. 6:**
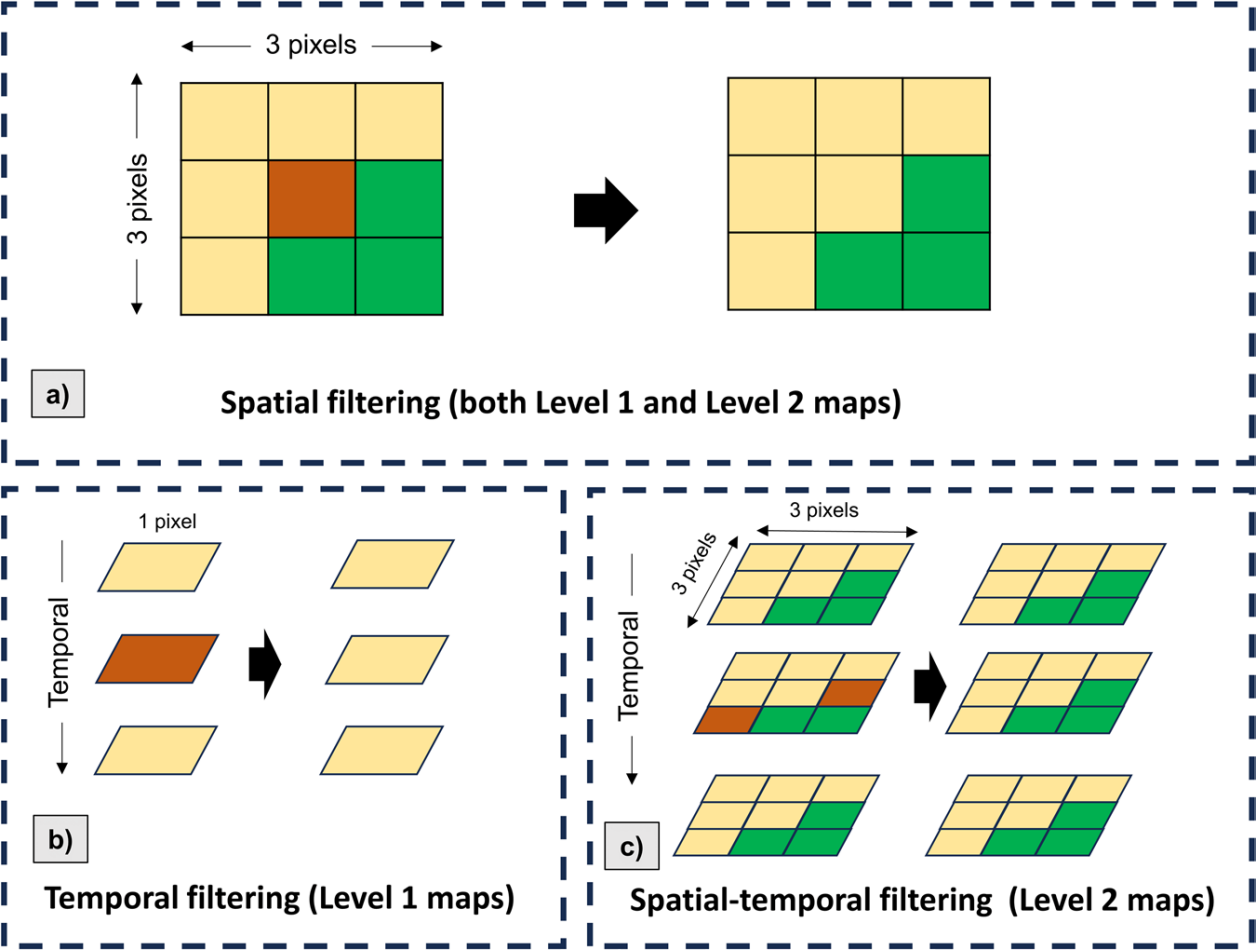
Post processing. (**a**) Spatial filtering (applying to both Level 1 and Level 2 maps); (**b**) Temporal filtering (for Level 1 maps); (**c**) Spatial-temporal filtering (for Level 2 maps).

We performed post-processing for maps for: Level-1 and Level-2 maps (Fig. 6).

- For both levels, we applied spatial filtering independently for each product (Fig. 6a). Specifically, in each annual map, we used a 3 × 3 pixels majority filter across the map. For each run, if the center pixel’s LC class differed from the eight surrounding pixels, it was converted into the major LC class within the window.
- For Level 1 maps, after spatial filtering, we performed temporal filtering for every pixel (Fig. 6b). Here, the temporal window has a length of 3 (years) running backwards from 2022 to 2000. For each run, if the surrounding years’ pixels have the same LC and the center year’s pixel has different LC, the center year’s LC is converted into the surrounding LC.
- For Level 2 maps, the temporal filtering process cannot be applied to the maps since crop sequences can happen frequently, i.e., one crop type pixel can be changed to others in next year and returned to the same type in the following years. Hence, we applied a hybrid method namely spatial-temporal filtering (Fig. 6c). Here, a 3 × 3 × 3 pixels cube moving window (height × width × temporal) runs across maps of every three years simultaneously (backward from 2022 to 2000). In each current window, if two patches of the surrounding years have the same LC types in all nine pixels, the pixels of the center patch are converted to the pixels of the surrounding patches only if it also has at least seven similar pixel values. This method allows us to filter temporal noise up to two pixels in a 3 × 3 window, while ensuring that the crop sequences are not over-filtered.

**Fig. 7:**
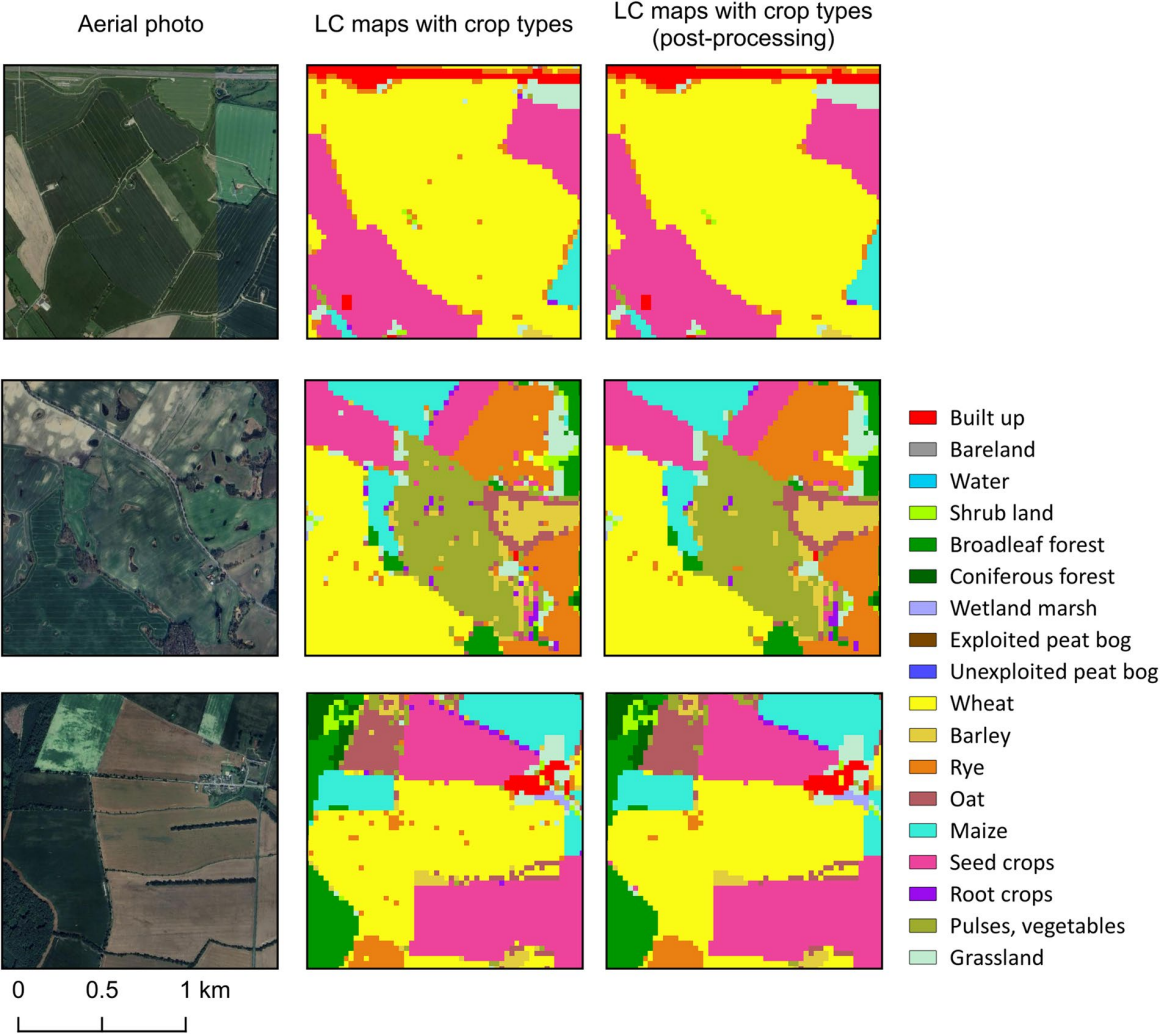
Examples of Level 2 land cover maps (2015) before and after post-processing.

The post-processing methods greatly improved the maps’ visuals by reducing noise (Fig. 7). To ensure the quality of the maps, we compared the accuracy of the maps before and after post-processing at each level. The related confusion matrices are shown in Supplement File 1.

## Data Records

The Baltic Sea Region Land Cover *Plus* (BSRLC+) dataset is available in the Zenodo repository^34^ (https://zenodo.org/records/10653871). The dataset consists of twenty-three annual land cover maps (from 2000 to 2022), containing 18 land cover types (see Fig. 1), in GeoTIFF format, with a 30 m x 30 m spatial resolution, projected to ETRS89-extended/LAEA Europe (EPSG:3035). The classification legend is shown in Table 4, and included as an additional file in the Zenodo repository (BSRLC_legend.xlss).

**Table 4:** BSRLC+ land cover types and corresponding values.

LC type	Map value	LC type	Map value
Built-up	1	Wheat	10
Bareland	2	Barley	11
Water	3	Rye	12
Shrubland	4	Oats	13
Coniferous forest	5	Maize	14
Broadleaf forest	6	Seed crops	15
Wetland marsh	7	Root crops	16
Exploited peat bog	8	Pulses, vegetables	17
Unexploited peat bog	9	Grassland	18

The training and validation data used in this study to create the maps are available in a separate Zenodo repository^35^ (https://zenodo.org/records/11073291). In the dataset, we provide point vector files in geopackage format (.gpkg) containing LC training and validation data. Each reference point is located in the center of a 30 × 30 m pixel. Data is projected to ETRS89-extended/LAEA Europe (EPSG:3035). The training points include general LC types which are considered to be consistent (invariant) during the period from 2006 to 2018. Training points for crop types are derived from EuroCrop dataset^30^, available for the three years: 2019, 2021 and 2023. For validation we used the manually annotated data (Table 5), as well as the harmonized version of the Land Use/Cover Area frame Survey (LUCAS) data^36^, which we reclassified to match the BSRLC+ legend (Table 4)

## Technical Validation

### Validation data

The Land Use/Cover Area frame Survey (LUCAS, https://ec.europa.eu/eurostat/web/lucas) program provides *in-situ* LULC. In this study, we used the harmonized version of LUCAS data^36^ available in four years (2009, 2012, 2015, 2018) to independently validate the BSRLC + maps.

Since LUCAS points are often annotated with the LC type of the exact surveying location, they do not always represent the LC of 30 m resolution pixels. Thus, we only selected LUCAS points based on the physical representation at 30 m resolution. This was performed by manual interpretation of the homogeneity of the pixels containing LUCAS points using high resolution imagery from Google Earth. For three classes, i.e., wetland marsh, exploited peat bog and unexploited peat bog, there were only a few samples from the LUCAS surveys (possibly due to the limited accessibilities to the surveying areas), and the LUCAS data also do not separate the peat bog classes. Hence, we performed manual labelling for these three specific classes. To do this, we used the original LUCAS grids (2 km × 2 km) combined with the Global Peatland Database (GPD, https://greifswaldmoor.de/global-peatland-database-en.html). The intersected LUCAS - GPD points were then manually labelled in four years (2009, 2012, 2015 and 2018) using historical high-resolution images from Google Earth. As a result, we acquired around 15,000 to 19,000 validation points in each year (2009, 2012, 2015 and 2018, see Table 5)

**Table 5:** Number of validation points in four years 2009, 2012, 2015 and 2018.

		2009	2012	2015	2018
Built-up		505	608	655	999
Bareland		65	88	78	63
Water		438	682	483	525
Shrubland		292	409	409	360
Broadleaf forest		1564	2230	2207	1518
Coniferous forest		4169	4912	4857	2908
Wetland	Wetland marsh	104	104	105	106
	Exploited peat bog	104	116	118	124
	Unexploited peat bog	665	665	665	663
Cropland	Wheat	1989	2098	2697	2648
	Barley	1121	1273	1154	1304
	Rye	670	710	722	722
	Oat	361	511	388	411
	Maize	571	1011	1076	1212
	Seed crops	888	865	1162	1264
	Root crops	432	523	507	633
	Dry pulse, vegetable	111	150	271	308
Grassland		1781	1664	1830	1688
Total		15830	18619	19384	16938

### Baseline (9-classes LC maps) assessments

First, we evaluated the thematic accuracies of our maps by comparing them to existing LULC products. We compared our BSRLC+ maps to CORINE land cover (CLC^2^, 100 m resolution, available in 2012 and 2018), Continental-European land cover (P-ELC^6^, available in 2009, 2012, 2015 and 2018) and Pan-European land cover (P-ELC^3^, available in 2015). To create comparable results, we aggregated maps of all products into nine LC classes. Specifically, all crop classes (excluding grassland) were aggregated as Cropland class; three classes: Wetland marsh, exploited peat bog and unexploited peat bog were aggregated as Wetland. Subsequently, we measured the F1 score for each class, with:

1 $$F1=\frac{\sum {True\; Positive}}{\sum {True\; Positive}+0.5\left(\sum {True\; Positive}+\sum {False\; Positive}\right)}$$

The baseline validation results (Table 6) showed that our maps produced the highest scores in every class compared to the three other LC products^2,3,6^. Notably, both P-ELC^3^ and C-ELC^6^ maps were created by supervised models that were trained with the *in-situ* LUCAS data directly. Our classifiers, on the other hand, were trained with independent datasets, and yet achieved the best validation results with LUCAS data. Hence, the thematic accuracy of our maps for nine LC types fully satisfies the standard of existing LULC maps in Europe.

**Table 6:** Class-wise F1-score of 4 LULC products.

		BSRLC+	CLC^2^	C-ELC^6^	P-ELC^3^
Buit-up	2009	0.96		0.90	
	2012	0.96	0.83	0.90	
	2015	0.97		0.90	0.93
	2018	0.98	0.87	0.91	
Bareland	2009	0.69		0.59	
	2012	0.70	0.54	0.58	
	2015	0.84		0.65	0.58
	2018	0.67	0.34	0.54	
Water	2009	1.0		0.98	
	2012	0.99	0.95	0.98	
	2015	0.99		0.98	0.97
	2018	1.0	0.94	0.98	
Shrubland	2009	0.36		0.20	
	2012	0.42	0.16	0.21	
	2015	0.51		0.27	0.50
	2018	0.55	0.13	0.15	
Broadleaf forest	2009	0.88		0.80	
	2012	0.90	0.61	0.80	
	2015	0.91		0.87	0.90
	2018	0.91	0.62	0.86	
Coniferous forest	2009	0.95		0.94	
	2012	0.95	0.86	0.93	
	2015	0.96		0.95	0.96
	2018	0.96	0.84	0.93	
Wetland	2009	0.91		0.88	
	2012	0.90	0.82	0.88	
	2015	0.93		0.91	0.91
	2018	0.95	0.83	0.90	
Cropland	2009	0.96		0.92	
	2012	0.97	0.91	0.94	
	2015	0.98		0.95	0.98
	2018	0.97	0.92	0.94	
Grassland	2009	0.83		0.75	
	2012	0.84	0.59	0.80	
	2015	0.87		0.81	0.87
	2018	0.85	0.49	0.77	

### Full (18-classes LC maps) assessments

Next, we evaluated accuracies of all 18-classes of the BSRLC+ maps in four years (2009 - Table 7, 2012 - Table 8, 2015 - Table 9, and 2018 - Table 10). Here, for each class, we measured the mapped area and estimated area (in km^2^), Overall Accuracy (OA), Producer Accuracy (PA) and User Accuracy (UA) using the validation procedure of Olofsson *et al*.^37^. This approach takes the total mapped areas of each LC into consideration and provides the uncertainty of each metric with confidence intervals.

**Table 7:** Accuracy assessments in 2009.

LC name	Mapped area (km^2^)	Estimated area (km^2^)	Producer’s accuracy	User’s accuracy
Built-up	45732	44364 ± 1287	0.98 ± 0.02	0.95 ± 0.02
Bareland	2901	5058 ± 1748	0.39 ± 0.14	0.68 ± 0.11
Water	203698	201855 ± 1800	1.00 ± 0.00	0.99 ± 0.01
Shrubland	84162	39537 ± 4291	0.65 ± 0.05	0.30 ± 0.04
Broadleaf forest	128648	135297 ± 3906	0.84 ± 0.02	0.88 ± 0.02
Coniferous forest	223822	257119 ± 4430	0.84 ± 0.01	0.97 ± 0.01
Wetland marsh	25194	10682 ± 1859	0.92 ± 0.09	0.39 ± 0.06
Exploited peat bog	998	2294 ± 949	0.42 ± 0.17	0.96 ± 0.04
Unexploited peat bog	6340	18249 ± 2688	0.34 ± 0.05	0.97 ± 0.01
Wheat	74779	83733 ± 2952	0.61 ± 0.02	0.68 ± 0.02
Barley	29696	47342 ± 2530	0.35 ± 0.02	0.56 ± 0.03
Rye	35462	32470 ± 2222	0.49 ± 0.03	0.45 ± 0.04
Oat	27151	16728 ± 1739	0.41 ± 0.06	0.25 ± 0.03
Maize	23877	26903 ± 1497	0.73 ± 0.04	0.83 ± 0.03
Seed crop	28071	29915 ± 1371	0.77 ± 0.03	0.82 ± 0.03
Root crop	5633	16085 ± 1419	0.31 ± 0.03	0.90 ± 0.04
Dry pulse, vegetable	8110	4863 ± 929	0.34 ± 0.10	0.21 ± 0.05
Grassland	174614	156392 ± 4021	0.89 ± 0.01	0.79 ± 0.02
Overall accuracy (%)	0.80 ± 0.01

**Table 8:** Accuracy assessments in 2012.

LC name	Mapped area (km^2^)	Estimated area (km^2^)	Producer’s accuracy	User’s accuracy
Built-up	43932	41488 ± 901	0.99 ± 0.01	0.93 ± 0.02
Bareland	2785	4103 ± 1030	0.50 ± 0.13	0.74 ± 0.10
Water	203519	201967 ± 1453	1.00 ± 0.00	0.99 ± 0.01
Shrubland	89796	53221 ± 4688	0.67 ± 0.04	0.40 ± 0.05
Broadleaf forest	135262	144906 ± 3590	0.85 ± 0.02	0.91 ± 0.01
Coniferous forest	21843	246565 ± 4241	0.85 ± 0.01	0.96 ± 0.01
Wetland marsh	26560	11617 ± 1979	0.88 ± 0.09	0.39 ± 0.06
Exploited peat bog	1079	2115 ± 843	0.49 ± 0.20	0.96 ± 0.04
Unexploited peat bog	7583	17305 ± 2470	0.42 ± 0.06	0.96 ± 0.01
Wheat	60457	74478 ± 2682	0.55 ± 0.02	0.68 ± 0.02
Barley	21660	47034 ± 2447	0.25 ± 0.02	0.54 ± 0.04
Rye	25535	28675 ± 1960	0.42 ± 0.03	0.48 ± 0.04
Oat	51468	20610 ± 1789	0.53 ± 0.05	0.21 ± 0.02
Maize	33244	38542 ± 1705	0.71 ± 0.03	0.82 ± 0.03
Seed crop	25836	27700 ± 1296	0.75 ± 0.03	0.81 ± 0.03
Root crop	8060	17367 ± 1358	0.34 ± 0.03	0.74 ± 0.05
Dry pulse, vegetable	11679	6157 ± 1038	0.32 ± 0.09	0.17 ± 0.04
Grassland	161994	145036 ± 3966	0.89 ± 0.02	0.80 ± 0.02
Overall accuracy (%)	0.79 ± 0.01

**Table 9:** Accuracy assessments in 2015.

LC name	Mapped area (km^2^)	Estimated area (km^2^)	Producer’s accuracy	User’s accuracy
Built-up	46281	44292 ± 768	0.99 ± 0.00	0.95 ± 0.02
Bareland	2613	3578 ± 960	0.64 ± 0.17	0.88 ± 0.08
Water	203912	201410 ± 1991	1.00 ± 0.00	0.99 ± 0.01
Shrubland	110286	65553 ± 5481	0.81 ± 0.03	0.48 ± 0.05
Broadleaf forest	126300	142118 ± 4123	0.82 ± 0.02	0.93 ± 0.01
Coniferous forest	216730	241204 ± 4540	0.87 ± 0.02	0.97 ± 0.01
Wetland marsh	17583	10642 ± 2030	0.82 ± 0.12	0.50 ± 0.07
Exploited peat bog	874	1840 ± 868	0.47 ± 0.22	0.98 ± 0.03
Unexploited peat bog	6089	16027 ± 2774	0.36 ± 0.06	0.96 ± 0.02
Wheat	76062	83939 ± 2153	0.73 ± 0.02	0.81 ± 0.01
Barley	29073	39550 ± 1899	0.49 ± 0.02	0.66 ± 0.03
Rye	31532	29418 ± 1665	0.65 ± 0.03	0.60 ± 0.04
Oat	33554	16362 ± 1574	0.49 ± 0.06	0.24 ± 0.03
Maize	32466	38611 ± 1314	0.77 ± 0.02	0.92 ± 0.02
Seed crop	31453	32530 ± 1113	0.83 ± 0.02	0.85 ± 0.02
Root crop	11159	14720 ± 1099	0.59 ± 0.04	0.78 ± 0.04
Dry pulse, vegetable	22167	12096 ± 1198	0.77 ± 0.05	0.42 ± 0.05
Grassland	130753	134997 ± 3381	0.86 ± 0.02	0.89 ± 0.01
Overall accuracy (%)	0.83 ± 0.01

**Table 10:** Accuracy assessments in 2018.

LC name	Mapped area (km^2^)	Estimated area (km^2^)	Producer’s accuracy	User’s accuracy
Built-up	46635	46534 ± 1138	0.97 ± 0.02	0.97 ± 0.01
Bareland	2811	3834 ± 964	0.59 ± 0.14	0.80 ± 0.12
Water	203573	203186 ± 785	1.00 ± 0.00	1.00 ± 0.00
Shrubland	117213	76460 ± 6417	0.86 ± 0.02	0.56 ± 0.05
Broadleaf forest	122920	132471 ± 4399	0.85 ± 0.03	0.92 ± 0.01
Coniferous forest	216296	232708 ± 4749	0.90 ± 0.02	0.96 ± 0.01
Wetland marsh	20382	12383 ± 1704	0.95 ± 0.06	0.58 ± 0.08
Exploited peat bog	1117	1576 ± 421	0.70 ± 0.19	0.99 ± 0.02
Unexploited peat bog	6229	19840 ± 3759	0.30 ± 0.06	0.96 ± 0.02
Wheat	72428	78996 ± 2307	0.66 ± 0.02	0.72 ± 0.02
Barley	40016	42117 ± 1882	0.56 ± 0.02	0.59 ± 0.03
Rye	29713	25960 ± 1535	0.62 ± 0.03	0.54 ± 0.04
Oat	28877	15131 ± 1401	0.52 ± 0.05	0.27 ± 0.03
Maize	26692	42649 ± 1819	0.57 ± 0.02	0.91 ± 0.02
Seed crop	29524	32546 ± 1232	0.75 ± 0.03	0.83 ± 0.02
Root crop	11070	18404 ± 1482	0.53 ± 0.04	0.88 ± 0.03
Dry pulse, vegetable	24272	11714 ± 1149	0.73 ± 0.05	0.35 ± 0.04
Grassland	129119	132378 ± 4478	0.84 ± 0.02	0.86 ± 0.02
Overall accuracy (%)	0.83 ± 0.01			

The OAs were relatively similar in the four validation years, with roughly 0.8 in 2009 and 2012, and 0.83 in 2015 and 2018, respectively. Class-wise, built-up areas, water, broadleaf forests, and coniferous forests consistently achieved the highest accuracies among all land cover classes, with both PAs and UAs consistently exceeding 0.8 across all validation years. Bareland showed PAs ranging from 0.50 to 0.64, with higher UAs ranging from 0.68 to 0.80. Shrubland exhibited slightly higher PAs than UAs, ranging from 0.65 to 0.86 and 0.30 to 0.56, respectively. Exploited and unexploited peat bogs typically had PAs between 0.3 and 0.4, except for 2018 when exploited peat bogs had a PA of 0.7, while both classes achieved UAs consistently above 0.9. Wetland marshes showed PAs ranging from 0.84 to 0.95, with UAs approximately between 0.39 and 0.58.

Results for agricultural classes varied considerably. Wheat, maize, seed crops, and grasslands consistently achieved higher accuracies than other classes across most years, with PAs and UAs ranging from 0.55 to 0.89 and 0.68 to 0.91, respectively. Other cereals such as barley, rye, and oats yielded PAs between 0.25 and 0.65 and UAs between 0.25 and 0.68. Root crops, dry pulses, and vegetables had the lowest accuracies initially, with PAs around 0.3 for both in 2009 and 2012, which notably improved to approximately 0.53 to 0.77 in 2015 to 2018. While root crops consistently achieved over 0.7 for UAs in all four years, the accuracy for dry pulses, and vegetable was notably lower, hovering around 0.2 in 2009 and 2012, and approximately 0.4 in 2015 and 2018. Overall, most agricultural classes displayed improved accuracies in 2015 and 2018 compared to earlier years.

### Comparison with crop statistics

We further evaluated the crop type classification by comparing the estimated areas with official agricultural statistics. Here, we used the national agricultural statistics data for Denmark^38^, which was available for 14 consecutive years from 2009 to 2022.

**Fig. 8:**
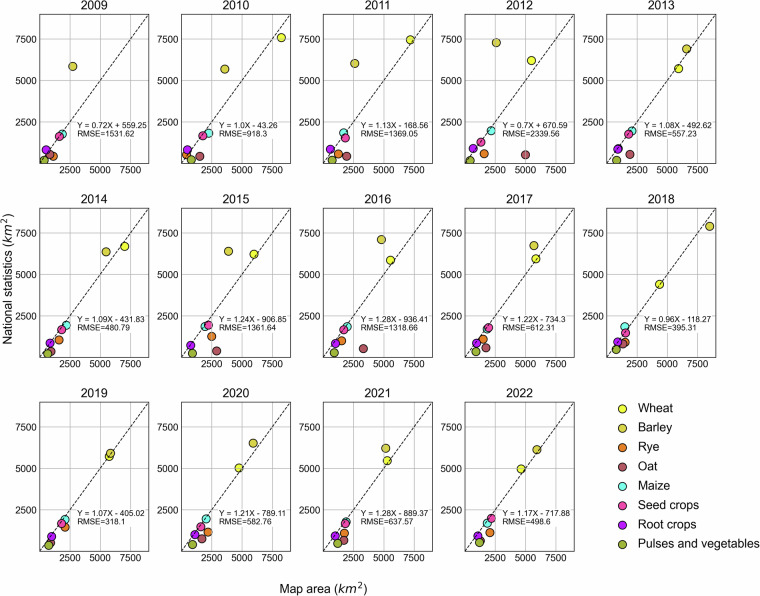
Annual crop statistics of Denmark from 2009 to 2022 compared to estimated areas from BSRLC+.

The results are shown in Fig. 8. Overall, crop area estimations from the BSRLC+ maps showed similar results as the Denmark national statistics in most years. Wheat accounted for the major agricultural area in the country in most years, which was a similar result as from the maps. However, great underestimations of Barley can be seen e.g., in 2009, 2010, 2011, 2012. This could be related to the overestimations of Oat and Rye in the same years, which could be seen from the confusion matrices. In addition, after 2017, crop area estimations tend to be more accurate in recent years, due to the availability of Sentinel-2 data which greatly improved the temporal density of the time series (Fig. 3).

### Qualitative assessment of peat exploitation

**Fig. 9:**
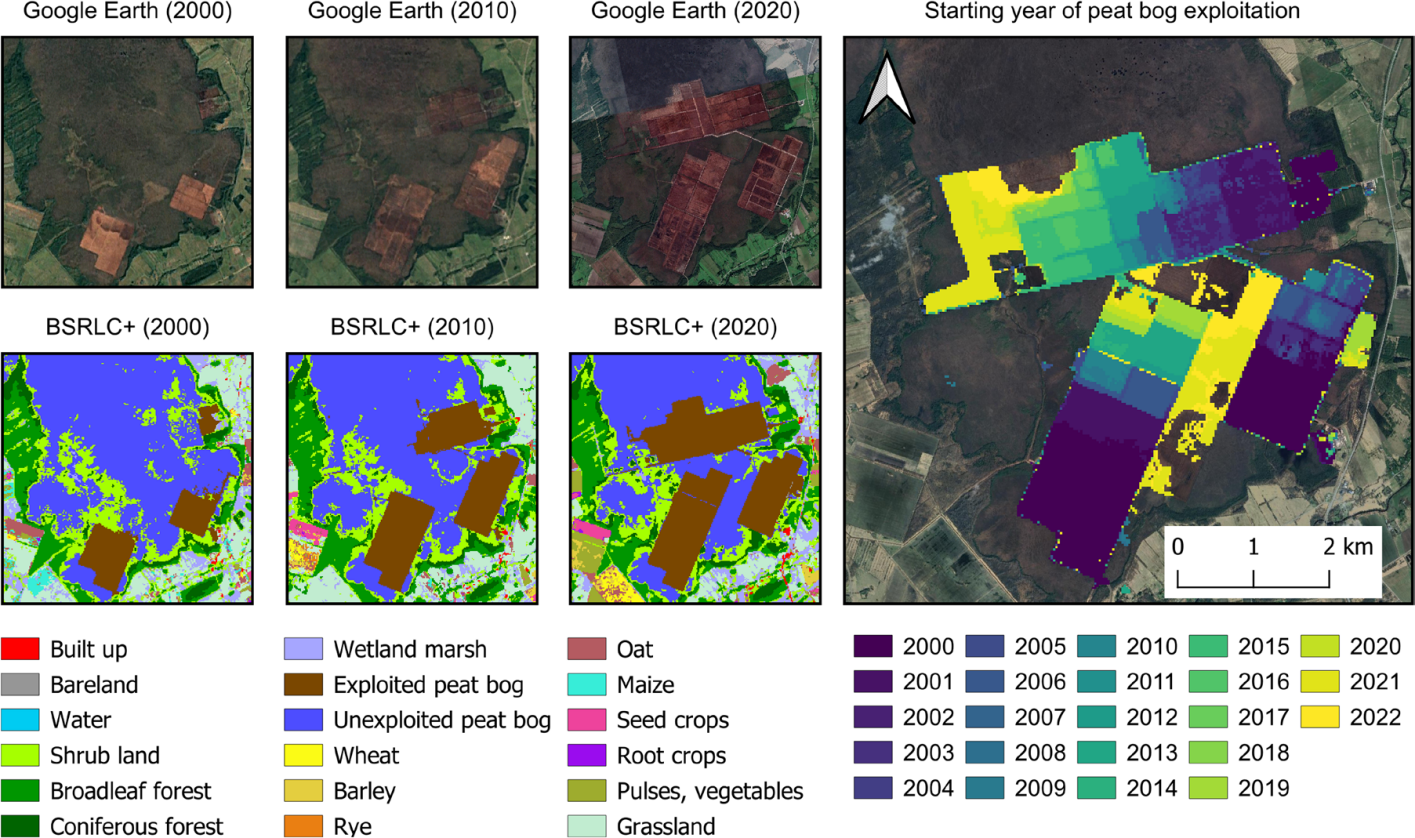
Peat bog exploitation in Estonia over two decades (2000–2022). (Left): Visual assessments showed similar patterns of exploited peatbog between high resolution images from Google Earth and the classification from BSRLC+ in three different years: 2000, 2010 and 2020. (Right): Estimated peatbog exploitation by year derived from the maps.

We evaluated the peat exploitation mapping quality using historical imageries from Google Earth. From the BSRLC + annual maps, we identified an area in Estonia (Fig. 9) where peat bogs have been actively mined every year from 2000 to 2022. From the high-resolution imageries, the exploited peat bogs appeared as linear trenches that can be visually distinguished from natural peat bogs in three years (2000, 2010 and 2020). In responses, our maps correctly captured the increases in mining areas in the respective years.

## Supplementary information

Supplement File 1

## Data Availability

The BSRLC+ maps are available in Zenodo repository^34^ (https://zenodo.org/records/10653871), training and validation datasets used in this study are available in a separate repository^35^ (https://zenodo.org/records/11073291). For creating the maps, we used open-source framework and tools to produce and present our mapping products, including Python 3.9, TensorFlow 2.10.0, QGIS 3.34. Remote sensing data was processed using FORCE, available on GitHub (https://github.com/davidfrantz/force). Codes used for land cover classification (including the pre-trained models) are available on GitHub (https://github.com/vudongpham/BSRLC).
