# Supplementary material for: An annual land cover dataset for the Baltic Sea Region with crop types and peat bogs at 30 m from 2000 to 2022
Source: Sci Data. 2024 Nov 18;11:1242. doi: 10.1038/s41597-024-04062-w (PMC11574315; doi:10.1038/s41597-024-04062-w)
Supplement: Supplementary file 1 — Supplement File 1 [file 41597_2024_4062_MOESM1_ESM.docx]

**Supplement information 1. Quantitative comparison before / after post-processing (LC maps)**

Table S1. F1 scores comparison (2009) before / after post-processing

|  | Before post-processing | After post-processing |
| --- | --- | --- |
| Built-up | 0.94 | 0.96 |
| Bareland | 0.73 | 0.70 |
| Water | 0.99 | 1.0 |
| Shrubland | 0.34 | 0.36 |
| Broadleaf forest | 0.87 | 0.88 |
| Coniferous forest | 0.95 | 0.95 |
| Wetland marsh | 0.46 | 0.54 |
| Exploited peat bog | 0.93 | 0.93 |
| Unexploited peat bog | 0.88 | 0.93 |
| Cropland and grassland | 0.98 | 0.99 |

Table S2. F1 scores comparison (2012) before / after postprocessing

|  | Before post-processing | After post-processing |
| --- | --- | --- |
| Built-up | 0.92 | 0.96 |
| Bareland | 0.61 | 0.7 |
| Water | 1.0 | 1.0 |
| Shrubland | 0.33 | 0.41 |
| Broadleaf forest | 0.88 | 0.9 |
| Coniferous forest | 0.94 | 0.95 |
| Wetland marsh | 0.35 | 0.54 |
| Exploited peat bog | 0.91 | 0.94 |
| Unexploited peat bog | 0.88 | 0.93 |
| Cropland and grassland | 0.97 | 0.99 |

Table S3. F1 scores comparison (2015) before / after postprocessing

|  | Before post-processing | After post-processing |
| --- | --- | --- |
| Built-up | 0.96 | 0.97 |
| Bareland | 0.81 | 0.85 |
| Water | 1.0 | 0.99 |
| Shrubland | 0.47 | 0.5 |
| Broadleaf forest | 0.91 | 0.91 |
| Coniferous forest | 0.96 | 0.96 |
| Wetland marsh | 0.53 | 0.62 |
| Exploited peat bog | 0.93 | 0.94 |
| Unexploited peat bog | 0.9 | 0.94 |
| Cropland and grassland | 0.99 | 0.99 |

Table S4. F1 scores comparison (2018) before / after postprocessing

|  | Before post-processing | After post-processing |
| --- | --- | --- |
| Built-up | 0.96 | 0.98 |
| Bareland | 0.59 | 0.67 |
| Water | 1.0 | 1.0 |
| Shrubland | 0.5 | 0.55 |
| Broadleaf forest | 0.91 | 0.91 |
| Coniferous forest | 0.95 | 0.96 |
| Wetland marsh | 0.61 | 0.7 |
| Exploited peat bog | 0.93 | 0.94 |
| Unexploited peat bog | 0.92 | 0.93 |
| Cropland and grassland | 0.99 | 0.99 |

**Supplement information 2. Quantitative comparison before / after post-processing (LC maps with crop types)**

Table S5. F1 scores comparison (2009) before / after postprocessing

|  | Before post-processing | After post-processing |
| --- | --- | --- |
| Built-up | 0.96 | 0.96 |
| Bareland | 0.71 | 0.69 |
| Water | 1.0 | 1.0 |
| Shrubland | 0.36 | 0.36 |
| Broadleaf forest | 0.88 | 0.88 |
| Coniferous forest | 0.95 | 0.95 |
| Wetland marsh | 0.54 | 0.54 |
| Exploited peat bog | 0.93 | 0.93 |
| Unexploited peat bog | 0.93 | 0.93 |
| Wheat | 0.68 | 0.68 |
| Barley | 0.46 | 0.46 |
| Rye | 0.46 | 0.46 |
| Oat | 0.33 | 0.33 |
| Maize | 0.79 | 0.79 |
| Seed crops | 0.83 | 0.82 |
| Root crops | 0.58 | 0.58 |
| Pulses, vegetables | 0.25 | 0.27 |
| Grassland | 0.83 | 0.83 |

Table S6. F1 scores comparison (2012) before / after postprocessing

|  | Before post-processing | After post-processing |
| --- | --- | --- |
| Built-up | 0.96 | 0.96 |
| Bareland | 0.73 | 0.70 |
| Water | 1.0 | 0.99 |
| Shrubland | 0.41 | 0.42 |
| Broadleaf forest | 0.9 | 0.9 |
| Coniferous forest | 0.95 | 0.95 |
| Wetland marsh | 0.54 | 0.53 |
| Exploited peat bog | 0.94 | 0.94 |
| Unexploited peat bog | 0.93 | 0.63 |
| Wheat | 0.65 | 0.65 |
| Barley | 0.4 | 0.40 |
| Rye | 0.42 | 0.43 |
| Oat | 0.31 | 0.30 |
| Maize | 0.76 | 0.77 |
| Seed crops | 0.81 | 0.80 |
| Root crops | 0.54 | 0.54 |
| Pulses, vegetables | 0.22 | 0.23 |
| Grassland | 0.84 | 0.84 |

Table S7. F1 scores comparison (2015) before / after postprocessing

|  | Before post-processing | After post-processing |
| --- | --- | --- |
| Built-up | 0.97 | 0.97 |
| Bareland | 0.85 | 0.84 |
| Water | 0.99 | 0.99 |
| Shrubland | 0.50 | 0.51 |
| Broadleaf forest | 0.91 | 0.91 |
| Coniferous forest | 0.96 | 0.96 |
| Wetland marsh | 0.62 | 0.63 |
| Exploited peat bog | 0.94 | 0.94 |
| Unexploited peat bog | 0.94 | 0.94 |
| Wheat | 0.80 | 0.80 |
| Barley | 0.59 | 0.60 |
| Rye | 0.59 | 0.59 |
| Oat | 0.32 | 0.31 |
| Maize | 0.84 | 0.85 |
| Seed crops | 0.85 | 0.86 |
| Root crops | 0.75 | 0.76 |
| Pulses, vegetables | 0.49 | 0.52 |
| Grassland | 0.87 | 0.87 |

Table S8. F1 scores comparison (2018) before / after postprocessing

|  | Before post-processing | After post-processing |
| --- | --- | --- |
| Built-up | 0.98 | 0.98 |
| Bareland | 0.70 | 0.67 |
| Water | 1.00 | 1.00 |
| Shrubland | 0.55 | 0.55 |
| Broadleaf forest | 0.91 | 0.91 |
| Coniferous forest | 0.96 | 0.96 |
| Wetland marsh | 0.70 | 0.70 |
| Exploited peat bog | 0.94 | 0.94 |
| Unexploited peat bog | 0.93 | 0.93 |
| Wheat | 0.72 | 0.72 |
| Barley | 0.59 | 0.59 |
| Rye | 0.56 | 0.55 |
| Oat | 0.34 | 0.35 |
| Maize | 0.71 | 0.72 |
| Seed crops | 0.82 | 0.82 |
| Root crops | 0.76 | 0.76 |
| Pulses, vegetables | 0.46 | 0.47 |
| Grassland | 0.85 | 0.85 |

**Supplement information 3. 1D-CNN network architecture**

To train deep learning models for land cover classification, we used Tensorflow version 2.10.1 ^1^.

- Con1D: 1D convolution filters that take two parameters (filter numbers, kernel size)

- ReLU: rectified linear unit activation with $ReLU\left( x \right)=\max\left( 0, x \right)= \left\{ \frac{x if x>0}{0 otherwise} \right.$

- Batch_Norm: Batch normalization ^2^

- Maxpooling: Max pooling operator for temporal data

- Flatten: Flattening the input data

- Dense: Fully connected filter that takes input and creates dense layer of (m) features

-Softmax: Softmax function with $Softmax{(Z)}_{i}=\frac{e^{Z_{i}}}{\sum_{j=1}^{K} e^{Z_{i}}} for i=1, \ldots, K and z=\left( z_{1}, \ldots, z_{K} \right)\in R$

- We used Adam optimizer ^3^ to optimize the loss-function (cross-entropy) during training

Table S9. 1D-CNN architecture.

| Input -> (52, 9) | |
| --- | --- |
| ReLU(Batch_Norm((Conv1D(128, 16))) -> (52, 128) | |
| ReLU(Batch_Norm((Conv1D(128, 16))) -> (52, 128) | |
| Maxpooling -> (26, 128) | |
| ReLU(Batch_Norm((Conv1D(256, 3))) -> (26, 256) | |
| ReLU(Batch_Norm((Conv1D(256, 3))) -> (26, 256) | |
| Maxpooling -> (13, 256) | |
| ReLU(Batch_Norm((Conv1D(512, 3))) -> (13, 512) | |
| ReLU(Batch_Norm((Conv1D(512, 3))) -> (13, 512) | |
| Level 1 mapping | Level 2 mapping |
| Dense -> (8) | Dense -> (11) |
| Softmax -> (8) | Softmax -> (11) |

**Reference:**

1 Martín, A. *et al.* TensorFlow: Large-Scale Machine Learning on Heterogeneous Systems. (2015).

2 Ioffe, S. & Szegedy, C. in *International conference on machine learning.* 448-456 (pmlr).

3 Kingma, D. P. & Ba, J. Adam: A method for stochastic optimization. *arXiv preprint arXiv:1412.6980* (2014).
